# Distinct Clinical Outcomes of Non-Small Cell Lung Cancer Patients with Epidermal Growth Factor Receptor (*EGFR*) Mutations Treated with *EGFR* Tyrosine Kinase Inhibitors: Non-Responders versus Responders

**DOI:** 10.1371/journal.pone.0083266

**Published:** 2013-12-23

**Authors:** Shih-Hsin Hsiao, H. Eugene Liu, Hsin-Lun Lee, Chii-Lan Lin, Wei-Yu Chen, Zhung-Han Wu, Sey-En Lin, Ling-Ling Chiang, Chi-Li Chung

**Affiliations:** 1 Division of Pulmonary Medicine, Department of Internal Medicine, Taipei Medical University Hospital, Taipei, Taiwan; 2 Division of Hematology and Oncology, Department of Internal Medicine, Wan Fang Hospital, Taipei Medical University, Taipei, Taiwan; 3 Department of Radiation Oncology, Wan Fang Hospital, Taipei Medical University, Taipei, Taiwan; 4 Division of Pulmonary Medicine, Department of Internal Medicine, Shuang Ho Hospital, Taipei Medical University, Taipei, Taiwan; 5 Department of Pathology, Wan Fang Hospital, Taipei Medical University, Taipei, Taiwan; 6 Department of Pathology, Taipei Medical University Hospital, Taipei, Taiwan; 7 School of Respiratory Therapy, College of Medicine, Taipei Medical University, Taipei, Taiwan; Shanghai Jiao Tong University School of Medicine, China

## Abstract

**Introduction:**

Treatment with epidermal growth factor receptor (*EGFR*) tyrosine kinase inhibitors (TKIs) has been associated with favorable progression free survival (PFS) in patients with non-small cell lung cancers (NSCLC) harboring *EGFR* mutations. However, a subset of this population doesn't respond to *EGFR*-TKI treatment. Therefore, the present study aimed to elucidate survival outcome in NSCLC *EGFR*-mutant patients who were treated with *EGFR* TKIs.

**Methods:**

Among the 580 consecutive NSCLC patients who were treated at our facility between 2008 and 2012, a total of 124 treatment-naïve, advanced NSCLC, *EGFR*-mutant patients treated with *EGFR* TKIs were identified and grouped into non-responders and responders for analyses.

**Results:**

Of 124 patients, 104 (84%) responded to treatment, and 20 (16%) did not; and the overall median PFS was 9.0 months. Notably, the PFS, overall survival (OS) and survival rates were significantly unfavorable in non-responders (1.8 *vs.* 10.3 months, hazard ratio (HR) = 29.2, 95% confidence interval (CI), 13.48–63.26, *P*<0.0001; 9.4 *vs.* 17.3 months, HR = 2.74, 95% CI, 1.52–4.94, *P* = 0.0008; and 58% *vs.* 82% in 6, 37% *vs.* 60% in 12, and 19 *vs.* 40% at 24 months, respectively). In multivariate analysis, treatment efficacy strongly affected PFS and OS, independent of covariates (HR = 47.22, 95% CI, 17.88–124.73, *P*<0.001 and HR = 2.74, 95% CI, 1.43–5.24, *P* = 0.002, respectively). However, none of the covariates except of the presence of *EGFR* exon 19 deletion in the tumors was significantly associated with better treatment efficacy.

**Conclusions:**

A subset of NSCLC *EGFR*-mutant patients displayed unfavorable survival despite *EGFR* TKI administration. This observation reinforces the urgent need for biomarkers effectively predicting the non-responders and for drug development overcoming primary resistance to *EGFR* TKIs. In addition, optimal therapeutic strategies to prolong the survival of non-responders need to be investigated.

## Introduction

Lung cancer, which is the most common cause of cancer deaths worldwide, is generally associated with poor prognoses. Recently, advances in personalized medicine have modestly improved treatment efficacy, toxicity and survival in subsets of lung cancer patients. Epidermal growth factor receptor (*EGFR*) mutation status has been shown to be significantly associated with tumor response to *EGFR* tyrosine kinase inhibitors (TKIs)[1], [2], leading to the routine assessment of the presence of *EGFR* mutations in advanced non-small cell lung cancers (NSCLC), particularly adenocarcinomas[3], [4]. Furthermore, *EGFR* TKIs have been recommended as first-line treatment for patients with advanced NSCLC that contain *EGFR* mutations due to the clinical benefits of these novel anti-tumor agents.

Prospective clinical trials have clearly demonstrated that *EGFR* TKIs are effective therapeutics that carry a 60–82% response rate[2], [5]–[7] and improve progression-free survival (PFS) with 7.7–13.3 months in NSCLC *EGFR*-mutant patients[2], [5]–[7]. However, 20–40% of NSCLC patients do not experience tumor reduction following *EGFR* TKI administration despite the presence of *EGFR* mutations in their tumors. This issue has not been well addressed. Specifically, PFS in NSCLC *EGFR*-mutant patients whose tumors do not significantly shrink after targeted therapy is rarely reported, contributing to the lack of comprehensive information about the treatment outcome of this subset of NSCLC patients.

In the present study, we aimed to determine survival outcome in treatment-naïve NSCLC patients whose tumors harbored *EGFR* mutations and who were treated with *EGFR* TKIs as first-line therapy, with a focus on comparing non-responders to responders.

## Materials and Methods

### Case Identification

We retrospectively reviewed the medical records of 580 consecutive patients who were histologically or cytologically diagnosed of NSCLC, including adenocarcinoma, squamous cell carcinoma (SCC) or NSCLC not otherwise specified (NOS), and treated at Taipei Medical University Hospital between January 2008 and November 2012, with an approval from the Joint Institutional Review Board (JIRB) of Taipei Medical University, Taipei, Taiwan (Approval number: 201108006). Additionally, the JIRB also waived the need for written informed consent from the patients. Patients with NSCLC that harbored *EGFR* mutations and who received *EGFR* TKIs (either gefitinib or erlotinib) as front-line treatment for advanced (stage IIIb or IV) NSCLC were eligible for these analyses. Patients with NSCLC that did not harbor *EGFR* mutations or NSCLC in which the *EGFR* mutation status was uncertain were excluded from the analyses. A patient who had NSCLC that contained any mutations in exons 18–21 of the *EGFR* gene was defined as an *EGFR* mutant. Patients who had previously received chemotherapy, had taken *EGFR* TKIs for less than 14 days, did not receive follow-up imaging studies, such as chest tomography (CT) scans or chest films, during the period of *EGFR* TKI administration, or had more than 1 primary cancer were excluded from the study.

### Variables

Demographic and clinical characteristics, including gender, age at diagnosis of lung cancer diagnosis or recurrence (cutoff at 60 years), smoking status (never *vs.* former or current), subtype of NSCLC histology (adenocarcinoma, SCC, NSCLC-NOS), stage (3b *vs.* 4b), and subtype of *EGFR* exon 18–21 mutations were collected. Additionally, Eastern Cooperative Oncology Group (ECOG) performance status (PS) at *EGFR* TKI administration, and response to *EGFR* TKI treatment (responder *vs.* non-responder) were also collected. In this study, follow-up time, PFS and overall survival (OS) were calculated from the date of *EGFR* TKI administration to the last follow-up, to the date of disease progression, and the date of death or the last follow-up, respectively. Patients whose NSCLC did not progress at the last follow-up were censored at the date of their last contact with our institution.

### Assessment of Response (Efficacy)

Treatment effectiveness and disease progression were determined using RECIST criteria[8]. Patients who were either in complete remission or who displayed a partial response were categorized as responders, and those with either stable disease or disease that had progressed were categorized as non-responders.

### Statistical Analyses

Frequencies and descriptive statistics on demographic and clinical characteristics were obtained. PFS and OS were estimated using the Kaplan-Meier method and the difference in survival between the subgroups was compared using log-rank test. The association of demographic and clinical characteristics with PFS and OS was evaluated using univariate and multivariate Cox regression. The associated factors with treatment efficacy of *EGFR* TKI were identified by univariate and multivariate logistic regression. The result was presented as odds ratio (OR) for logistic regression or hazard ratio (HR) for Cox regression with their corresponding 95% confidence intervals (CI). All of the data analyses were conducted using SPSS software version 18 (SPSS Inc, Chicago, Illinois).

## Results

**Figure 1:**
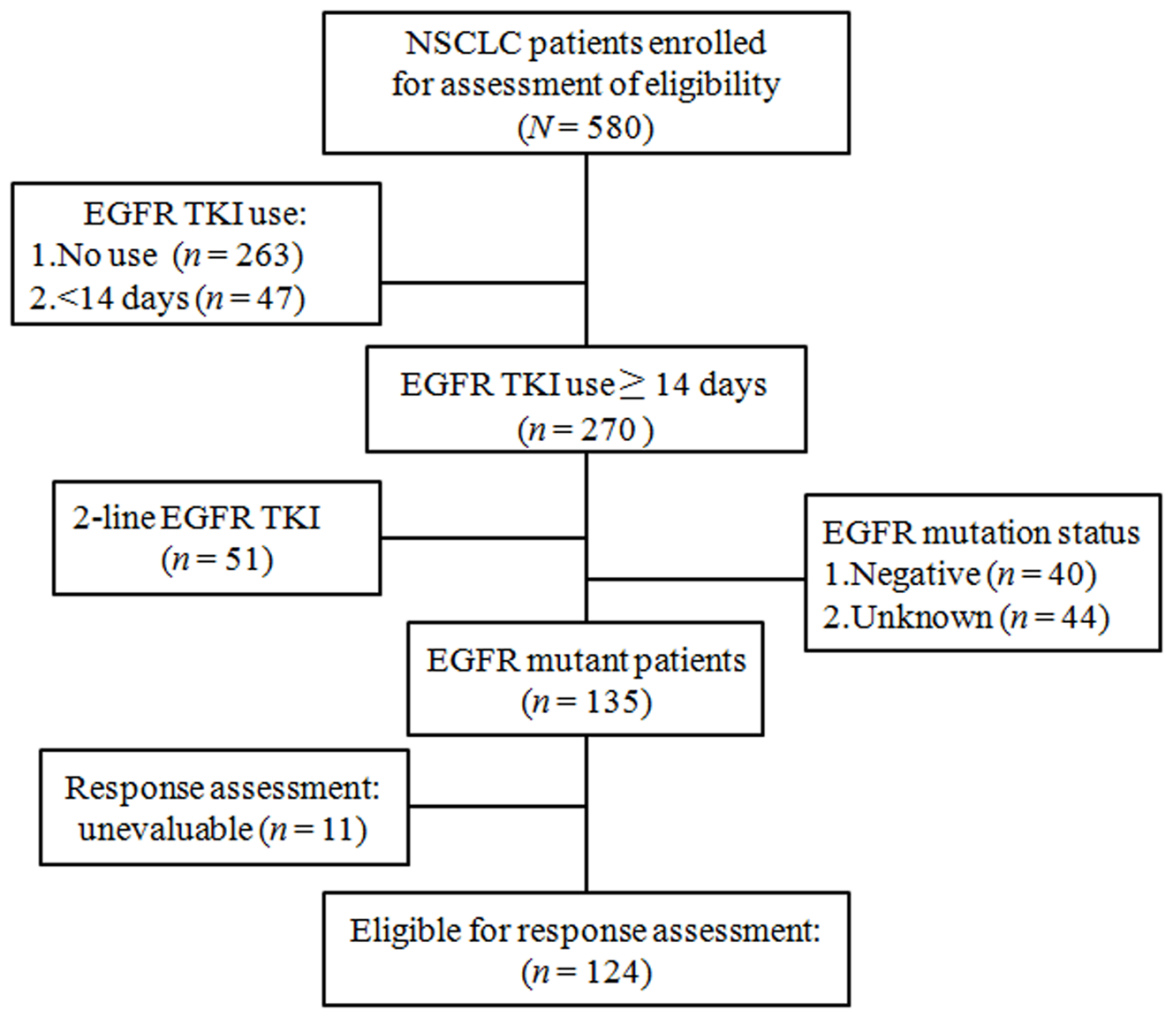
Flow chart of patients enrolled for analysis. NSCLC: non-small cell lung cancer, *EGFR*: epidermal growth factor receptor, TKI: tyrosine kinase inhibitor

A total of 124 NSCLC, including 121 adenocarcinoma, 2 NSCLC-NOS and 1 SCC, patients who received *EGFR* TKIs as the front-line treatment for their advanced NSCLC with *EGFR* mutations were identified for the analyses (Figure 1), with a mean age: 68.2±13.0 years, and median follow-up time: 9.8 months (inter-quartile rage  = 4.8–16.1 months). Sixty-three (50.8%) patients were alive at the last follow-up. The patient characteristics are listed in Table 1. Female gender, never smokers, and young age <60 years represented 66%, 79% and 23% of the patients, respectively. Moreover, the majority of eligible patients was at stage IV and displayed good performance status (ECOG PS 0 – 2). In addition, 92% of 124 patients possessed either gene deletion in *EGFR* exon 19 or point mutation in *EGFR* exon 21.

**Table 1:** Baseline characteristics of patients with NSCLC harboring *EGFR* mutations treated with *EGFR* TKIs.

Variables	All patients (N = 124)	Responder (n = 104)	Non-responder (n = 20)
Age (year)	68.2±13.0	68.7±13.0	65.6±12.9
<60	28 (23)	23 (22)	5 (25)
≥60	96 (77)	81 (78)	15 (75)
Gender			
Female	82 (66)	70 (67)	12 (60)
Male	42 (34)	34 (33)	8 (40)
Smoking history			
Never smoker	98 (79)	81 (78)	17 (85)
Current/former smoker	26 (21)	23 (22)	3 (15)
Stage at *EGFR* TKIs use			
3b	10 (8)	7 (7)	3 (15)
4	114 (92)	97 (93)	17 (85)
ECOG PS at treatment			
0–2	101 (81)	86 (83)	15 (75)
3–4	23 (19)	18 (17)	5 (25)
Histology			
Adenocarcinoma	121 (98)	101 (97)	20 (100)
SCC	2 (2)	2 (2)	0 (0)
NSCLC-NOS	1 (1)	1 (1)	0 (0)
Subtype of *EGFR* mutations			
exon 19	48 (39)	45 (43)	3 (15)
exon 21	66 (53)	53 (51)	13 (65)
exon 18 or 20	10 (8)	6 (6)	4 (20)

NSCLC: non-small cell lung cancer, NOS: not otherwise specified, *EGFR*: epidermal growth factor receptor, TKI: tyrosine kinase inhibitor, ECOG PS: eastern cooperation oncology group performance status, SCC: squamous cell carcinoma

The overall treatment efficacy of *EGFR* TKIs was 84% (104/124), and twenty patients (16%) did not experience significant tumor shrinkage despite *EGFR* TKI administration, as shown in Table 1. This observation implied that individuals with *EGFR* mutations in their tumors may not consistently experience clinical benefits, such as longer survival, following *EGFR* TKI treatment. Therefore, we compared PFS in non-responders to PFS in responders.

**Figure 2:**
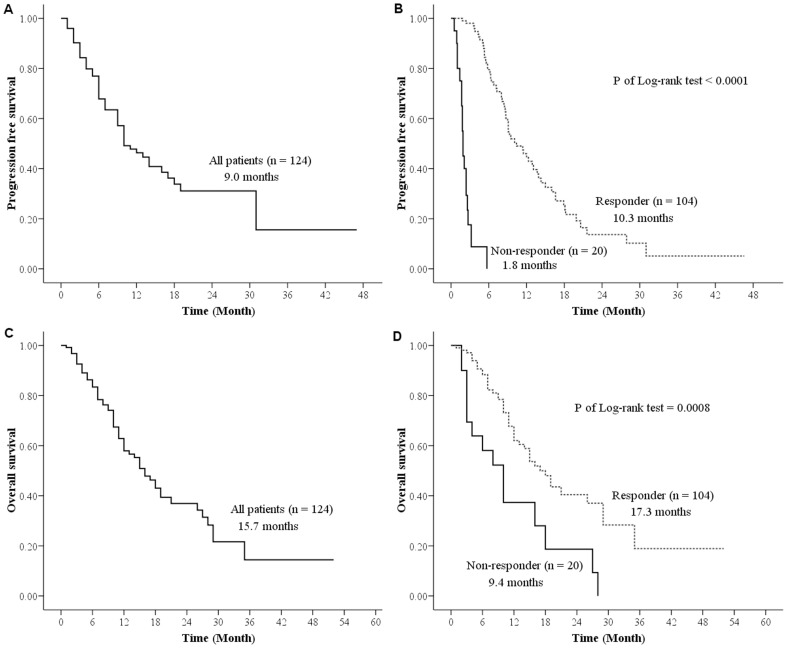
Progression-free survival A) in all, B) in patients stratified by non-responders and responders, and overall survival C) in all, D) in patients stratified by non-responders and responders.

The overall median PFS of 124 eligible patients was 9.0 months (25^th^ and 75^th^ percentiles were 16.5 and 5.2 months, respectively) (Figure 2A). Notably, PFS was significantly shorter in non-responders (1.8 *vs.* 10.3 months, HR = 29.2, 95%CI  = 13.48–63.26, *P*<0.0001) (Figure 2B). We further analyzed 101 patients with ECOG PS of 0–2 and found that the difference between the two groups remained significant (2.0 months in non-responders and 11.5 months in responders, *P*<0.0001). This analysis clearly demonstrated that a proportion of NSCLC *EGFR*-mutant patients did not display favorable PFS in spite of *EGFR* TKI administration.

Moreover, overall survival (OS) was 15.7 months (25^th^ and 75^th^ percentiles were 28.6 and 8.8 months, respectively) (Figure 2C) and was significantly poorer in non-responders (9.4 vs. 17.3 months, HR = 2.74, 95% CI = 1.52–4.94, *P* = 0.0008) (Figure 2D). Notably, the 6-, 12- and 24-month survival rates of non-responders were lower than responders (58% *vs.* 82%, 37% *vs.* 60%, and 19 *vs.* 40%, respectively). To confirm this observation, we further analyzed 101 patients with ECOG PS of 0–2 and found that OS was significantly poorer in non-responders (5.9 vs. 20.5 months, *P* = 0.0004). The survival discrepancy between these two groups may have been the result of differences in pre-treatment variables and tumor responses to *EGFR* TKIs in these patients.

To weigh the impact of baseline variables and treatment response on the subsequent survival outcome, multivariate analyses were conducted and revealed that response to *EGFR* TKIs was significantly associated with favorable PFS and OS, independent of age, performance status and subtype of *EGFR* mutation (HR = 47.22, 95% CI = 17.88–124.73, *P*<0.001, and HR = 2.74, 95% CI = 1.43–5.24, *P* = 0.002, respectively) as shown in Tables 2 and 3. These findings indicated that the treatment response could be translated into subsequent survival outcome, and suggested the importance of predictors of treatment efficacy for *EGFR* TKIs in NSCLC *EGFR*-mutant patients.

**Table 2:** Analysis of clinical variables associated with progression free survival in NSCLC *EGFR*-mutant patients.

	Univariate			Multivariate		
Predictors	HR	95% CI	*P*	HR	95% CI	*P*
Age ≥60 years	0.91	0.54–1.55	0.734	0.80	0.44–1.46	0.474
Male gender	1.10	0.70–1.75	0.681	1.02	0.57–1.85	0.942
Current/former smoker	1.07	0.64–1.79	0.799	1.20	0.62––2.33	0.586
Stage 4/recurrence	0.88	0.45–1.73	0.710	1.78	0.85–3.75	0.127
ECOG PS 3–4	1.66	0.98–2.81	0.062	1.98	1.12–3.50	0.019
Subtype of *EGFR* ‡						
exon 19	0.92	0.57–1.46	0.710	1.20	0.69–2.07	0.524
exon 18 or 20	2.03	0.90–4.55	0.087	2.50	1.08–5.81	0.033
TKI non-response	29.20	13.48–63.26	<0.001	47.22	17.88–124.73	<0.001

‡ reference group was exon 21. *EGFR*: epidermal growth factor receptor, TKI: tyrosine kinase inhibitor, ECOG PS: eastern cooperation oncology group performance status, HR: hazard ratio, CI: confidence interval.

**Table 3:** Analysis of variables associated with overall survival in NSCLC *EGFR*-mutant patients.

	Univariate			Multivariate		
Predictors	HR	95% CI	*P*	HR	95% CI	*P*
Age ≥60 years	1.30	0.69–2.46	0.413	1.23	0.62–2.44	0.556
Male gender	1.05	0.61–1.82	0.849	1.04	0.54–2.02	0.907
Current/former smoker	0.98	0.53–1.80	0.940	0.93	0.44–1.97	0.844
Stage 4/recurrence	0.89	0.42–1.90	0.768	1.11	0.49–2.49	0.804
ECOG PS 3–4	1.93	1.04–3.57	0.036	1.64	0.84–3.18	0.147
Subtype of *EGFR* ‡						
exon 19	0.63	0.36–1.10	0.107	0.82	0.44–1.50	0.515
exon 18 or 20	0.85	0.30–2.40	0.766	0.70	0.24–2.06	0.516
TKI non-response	2.74	1.52–4.94	0.001	2.74	1.43–5.24	0.002

‡ reference group was exon 21. *EGFR*: epidermal growth factor receptor, TKI: tyrosine kinase inhibitor, ECOG PS: eastern cooperation oncology group performance status, HR: hazard ratio, CI: confidence interval

To investigate the baseline clinical variables that could predict treatment response to *EGFR* TKIs, logistic regression models were used to determine the relationships between these factors and treatment response. In multivariate analyses (Table 4), the presence of *EGFR* exon 19 deletion in tumors was significantly associated with better treatment response (OR = 5.58, 95% CI = 1.30–23.93, *P* = 0.021), independent of the remaining clinical variables, including age, gender, history of smoking, stage, and performance status.

**Table 4:** Analysis of clinial variables associated with treatment efficacy of *EGFR* TKI in NSCLC *EGFR*-mutant patients.

	Univariate			Multivariate		
Predictors	OR	95% CI	*P*	OR	95% CI	*P*
Age ≥60 years	1.17	0.39–3.57	0.778	2.72	0.73–10.14	0.136
Male gender	0.73	0.27–1.95	0.528	0.37	0.11–1.24	0.108
Current/former smoker	1.61	0.43–5.97	0.477	2.01	0.44–9.11	0.365
Stage 4/recurrence	2.45	0.58–10.40	0.226	3.85	0.74–20.14	0.110
ECOG PS 3–4	0.63	0.20–1.95	0.421	0.59	0.17–2.06	0.411
Subtype of *EGFR* ‡						
exon 19	3.68	0.99–13.73	0.052	5.58	1.30–23.93	0.021
exon 18 or 20	0.37	0.09–1.50	0.162	0.34	0.08–1.57	0.169

‡ reference group was exon 21. *EGFR*: epidermal growth factor receptor, TKI: tyrosine kinase inhibitor, ECOG PS: eastern cooperation oncology group performance status, OR: odds ratio, CI: confidence interval

## Discussion

In the present study, we reconfirmed that a subset of NSCLC patients who were treated with *EGFR* TKIs did not experience marked tumor shrinkage despite the presence of *EGFR* mutations and clearly demonstrated that PS and OS strikingly differed in non-responders and responders (1.8 *vs.* 10.3 months, *P*<0.0001 and 9.4 *vs.* 17.3 months, *P* = 0.0008, respectively). These differences in PFS and OS mostly resulted from tumor response to treatment. However, pre-treatment clinical variables except the subtype of *EGFR* mutations failed to predict treatment efficacy. These findings emphasize the need for further studies investigating novel biomarkers that can predict *EGFR* TKI treatment efficacy, particularly the non-responders, and the primary mechanisms underlying resistance in NSCLC containing *EGFR* mutations.

The introduction of *EGFR* TKIs as first-line treatment has been generally accepted to improve tumor response rates and PFS in patients with advanced NSCLC harboring *EGFR* mutations compared to standard chemotherapy. Historically, PFS in this population has been reported to be approximately 7.7–13.3 months[2], [5]–[7]. In the present study, median PFS was 9.0 months for all eligible patients. It is reasonable to expect that the patients who did not experience significant tumor shrinkage after treatment would have unfavorable prognoses. However, to the best of our knowledge, PFS in non-responders, which accounted for a proportion (20–40%) of all of the enrolled patients in these breakthrough clinical trials[2], [5]–[7], has not been widely reported, contributing to the lack of information about the survival outcome of this subset who did not respond to *EGFR* TKI administration. In the present study, we did not intend to re-emphasize the overall advantages from *EGFR* TKI treatment in NSCLC *EGFR*-mutant patients compared to conventional chemotherapy. Instead, we focused on elucidating the distinct survival outcomes of non-responders and responders who were treated with *EGFR* TKIs as first-line treatment for their NSCLC with *EGFR* mutations.

In the present study, we demonstrated that PFS strikingly differed in non-responders and responders (1.8 *vs.* 10.3 months, *P*<0.0001) despite the fact that both groups possessed *EGFR* mutations and received *EGFR* TKIs as their first-line treatment. Moreover, our analyses revealed that OS differed significantly in both groups (9.4 *vs.* 17.3 months, *P* = 0.0008). These distinct survival outcomes in non-responders and responders prompted us to consider several critical issues that are encountered in clinical practice. For example, the process that should be used to select optimal candidates who possess *EGFR* mutations and will experience favorable tumor response to *EGFR* TKIs remains unclear. We do not know whether non-responders can be successfully identified prior to *EGFR* TKI administration or soon after treatment. Moreover, which regimen of cytotoxic chemotherapy is superior in non-responders when they are recognized before or after the start of *EGFR* TKI treatment, remain unknown.

The discrepancies in survival benefits following *EGFR* TKI administration among NSCLC *EGFR*-mutant patients may result from both differences in pre-treatment variables and tumor response to treatment. In our analyses, however, none of the baseline clinical factors was identified to be significantly associated with PFS and OS, with the exception of tumor response to TKIs, performance status and subtype of *EGFR* mutation. These findings repeatedly highlighted the importance of factors that can predict therapeutic efficacy. Disappointingly, neither demographic data nor baseline pre-treatment clinical factors was demonstrated to effectively discriminate non-responders from responders prior to treatment in the current analyses. Collectively, our findings revealed that clinical outcomes in NSCLC *EGFR*-mutant patients who were treated with *EGFR* TKIs were distinct, and imply that reproducible and reliable biomarkers that clearly predict treatment efficacy are crucial for the treatment of NSCLC *EGFR*-mutant patients. The development of novel drugs that can overcome primary *EGFR* TKI resistance is of equal importance.

Some pilot studies have reported that intrinsic factors in *EGFR*-mutant lung cancer cells, such as mutations in T790M, PI3CA, or KRAS and activation of the intracellular Fas/NF-κB signaling pathway, may confer primary resistance to *EGFR* TKIs[9]–[11]. In addition, exogenous factors from the tumor microenvironment, including hepatocyte growth factor and interleukin-6, may also play a role in primary *EGFR* TKI resistance[12], [13]. Furthermore, suboptimal drug exposure, which may result from dose-escalation due to toxicities, increased metabolism of *EGFR* TKIs by cytochrome P450 3A4, or cigarette smoke-induced upregulation of cytochrome P450 1A1, has been shown to be associated with primary drug resistance[14]. The results of these studies are promising, and may prove to be helpful in predicting therapeutic efficacy and overcoming resistance to TKIs in patients whose NSCLC harbors *EGFR* mutations in the near future.

There were several limitations of current study that may have influenced the analyses that were conducted. First, the number of non-responders was small (n = 20). However, the differences in PFS between responders and non-responders were marked. Thus, we believe that the major findings of this study will not be biased by the issue of patient number. Second, approximately 30% of the 124 enrolled patients displayed poor performance statuses (ECOG PS = 3–4), which may have influenced treatment efficacy. In an effort to exclude this possibility, multivariate analyses were conducted and revealed that performance status was not significantly associated with treatment efficacy, as shown in Table 4 (*P* = 0.411). We further focused on the 101 patients with ECOG PS of 0–2, and re-analyses indicated that the survival results were consistent with those in all eligible patients. In addition, 2^nd^-line treatment may have affected OS in responders and non-responders in this retrospective study, and we will address this issue by attempting to enroll more non-responders in the future.

In conclusion, we demonstrated that a subset of NSCLC *EGFR*-mutant patients did not experience tumor shrinkage, leading to unfavorable PFS and OS. Moreover, none of the clinical variables that we assessed could be successfully used to predict *EGFR* TKI treatment efficacy in this population with the exception of subtype of *EGFR* mutations. These observations highlight the need for a novel biomarker that effectively discriminates non-responders from responders prior to or earlier after *EGFR* TKI administration. In addition, optimal therapeutic strategies to prolong the survival of non-responders need to be investigated, and the development of novel drugs that can overcome primary *EGFR* TKI resistance is of equal importance.
